# A Framework for Detecting False Data Injection Attacks in Large-Scale Wireless Sensor Networks

**DOI:** 10.3390/s24051643

**Published:** 2024-03-02

**Authors:** Jiamin Hu, Xiaofan Yang, Lu-Xing Yang

**Affiliations:** 1 School of Big Data & Software Engineering, Chongqing University, Chongqing 400044, China; jiaminhu@cqu.edu.cn; 2 College of Information Technology, Deakin University, Melbourne, VIC 3125, Australia; y.luxing@deakin.edu.au

**Keywords:** large-scale sensor networks, false data injection attacks, detection framework, distributed solution

## Abstract

False data injection attacks (FDIAs) on sensor networks involve injecting deceptive or malicious data into the sensor readings that cause decision-makers to make incorrect decisions, leading to serious consequences. With the ever-increasing volume of data in large-scale sensor networks, detecting FDIAs in large-scale sensor networks becomes more challenging. In this paper, we propose a framework for the distributed detection of FDIAs in large-scale sensor networks. By extracting the spatiotemporal correlation information from sensor data, the large-scale sensors are categorized into multiple correlation groups. Within each correlation group, an autoregressive integrated moving average (ARIMA) is built to learn the temporal correlation of cross-correlation, and a consistency criterion is established to identify abnormal sensor nodes. The effectiveness of the proposed detection framework is validated based on a real dataset from the U.S. smart grid and simulated under both the simple FDIA and the stealthy FDIA strategies.

## 1. Introduction

Wireless sensor networks (WSNs) consist of spatially dispersed sensors connected via wireless communication protocols [1]. These sensors are equipped with sensing capabilities to collect data on environmental parameters and physical quantities, which are transmitted to a central server or data center for further analysis and decision-making. WSNs are widely employed in various fields, including military affairs, agriculture, healthcare, industrial automation, and intelligent transportation [2].

Typically, the sensors in WSNs are resource-constrained devices in unprotected environments that are vulnerable to physical tampering [3,4,5]. The behavior of an attacker who physically tampers with sensor data is known as a false data injection attack (FDIA). As a result of the FDIA, the tampered sensors provide misleading data to the central server, leading the system to make incorrect judgments. The FDIA undermines the authenticity of sensor data, which can seriously impact systems that rely on sensor data for decision-making or monitoring, culminating in economic loss or even a life crisis. As a result, it is critical to develop detection mechanisms to ensure that WSNs are resistant to FDIAs [6,7].

### 1.1. Motivation

The focus of this paper is on detecting FDIAs in large-scale WSNs. Our goal is to provide a detection framework for FDIAs with the following properties:

- **Stealthy FDIA detection**. The attacker’s purpose is to use resourceful and sophisticated strategies to minimize the risk of being identified. Stealthy FDIAs may be employed, i.e., making the injected false data look as close to the genuine data as possible, such as by mimicking genuine data distributions and time series patterns. Since stealthy FDIAs are typically not easily observed, the detection framework should take this into account to reduce the likelihood of potential harm.
- **Distribute detection**. The detection process might be centralized or distributed. In centralized detection, all sensor data are sent to a central node for thorough processing. In distributed detection, sensor data are evaluated separately by local sensors or edge devices, making it more responsive to data changes than centralized detection. More significantly, given the widely dispersed sensors and enormous data volumes in large-scale WSNs, distributed detection might be more straightforward to scale.
- **General detection**. Large-scale WSNs are employed in various fields, and the physical behavior of such systems is diverse. Electric power systems, for example, can be defined using circuit equations, whereas thermodynamic systems can be represented using thermodynamic laws. Therefore, a detection framework based only on a measurement that does not require domain-specific a priori knowledge is necessary, which makes the detection method more general and allows for similar detection methods to be applied to sensors in different domains without much adaptation.

### 1.2. Main Contributions

We propose a correlation-based framework for detecting FDIAs, and our main contributions are sketched below.

- We first develop a grouping approach based on the temporal correlation of the cross-correlation between the time-series signals of pairwise sensors. All sensors are categorized into multiple correlated groups, and subsequent detection methods are performed separately within the groups.
- We build an autoregressive integrated moving average (ARIMA) model for predicting future data from each sensor using historical time-series signals, which is used to learn the normal temporal correlation of the cross-correlation between data reported by pairwise sensors.
- Based on the comparison of the normal and actual temporal correlation of the cross-correlation within each group, the basis for determining the consistency of the pairwise sensor data is established. Then, majority voting is executed within each group to identify the abnormal sensors.
- To verify the performance of the detection framework, we construct simple FDIAs and stealthy FDIAs in a genuine sensor dataset. The effectiveness of our proposed detection framework is verified through extensive simulation experiments.

The subsequent materials are organized in this fashion: Section 2 reviews the related works. In Section 3, sensor data and correlation definitions are introduced. The detection framework is described in detail in Section 4, and the performance of the detection framework is corroborated through simulation experiments in Section 5. Finally, this work is summarized in Section 6.

## 2. Related Work

In this section, we make comments on the previous work that is related to the present paper, aiming to highlight the novelty of our work. Detecting FDIAs in sensors has received considerable attention. In this section, we categorize the existing related works into three research directions related to FDIA detection: FDIA detection methods and FDIA types.

### 2.1. FDIA Detection Methods

Recent studies have been conducted to detect FDIAs on sensors by modeling the physical behavior of the system. In general, the physical behavior of the system is established based on physical equations (fluid dynamics, electromagnetic laws, etc.) to predict the sensor data, and then the predicted data are compared to the actual data [8]. Some attempts have been made to build predictive models to detect FDIAs through the dynamical equations of smart grids [9,10], unmanned aerial vehicles [11], water distribution systems [12], and cyber–physical systems [13,14,15]. However, this detection approach requires appropriate predictive models for specific domains and relies on a priori knowledge of specific physical behaviors, which allows for limited scalability.

Subsequent studies have explored techniques for detecting FDIAs from sensor measurements, with the majority of these works based on exploring inter-measurement correlations. Illiano et al. [16] presented an approach to detecting FDIAs in WSNs that combines measurement checks and authentication strategies. Aboelwafa et al. [17] addressed an approach to detecting FDIAs in the industrial Internet of Things that exploits sensor data correlation in time and space. Martovytskyi et al. [18] explored the method of FDIA detection, which is based on spatiotemporal correlation in smart grids. Berjab et al. [19] presented a method for detecting FDIAs in WSNs, which uses observed spatiotemporal and multivariate attribute sensor correlations. Huang et al. [20] addressed the problem of detecting FDIAs in dynamic WSNs based on spatial correlation. Based on the spatiotemporal correlation, Hu et al. [21] explored the idea of fault diagnosis to detect collusive FDIAs in WSNs. However, these efforts depend on centralized detection, increasing the complexity and cost of detection systems in the face of increasing data volumes.

In contrast, distributed detection methods can be more easily scaled to large-scale sensor networks. Chen et al. [22] built distributed real-time detection algorithms based on spatiotemporal correlation to detect FDIAs in large-scale networked industrial sensing systems. Islam et al. [23] utilized distributed algorithms based on spatiotemporal correlation to detect data anomalies in large-scale intelligent transportation systems. Lai et al. [24] suggested a distributed approach to detecting FDIAs in WSNs using temporal, spatial, and event-based correlation. In this paper, our framework is based on a distributed approach, where detection methods can be executed at separate edge devices to reduce the network pressure associated with processing data generated by large-scale sensors.

### 2.2. FDIA Types

Another crucial consideration in FDIA detection is the type of attack. An adversary may employ simple attacks, such as randomly injecting high outliers and injecting false data with a common strategy. An adversary may employ stealthy attacks, such as constructing coherent attack signals. Most of the works [17,18,19,20,22,23,24] mentioned based on sensor measurements themselves are effective in detecting simple FDIAs, but not stealthy ones. For instance, in [22], based on the spatiotemporal correlation of sensor data, the authors used exponential weighted moving average and principal component analysis to establish a rotated ellipse area for each pair of sensors in a correlation group and detected FDIAs by determining whether the current sensor readings for each pair of sensors were located within the corresponding area of the rotated ellipse. Assuming that an attacker employs a collusive strategy whereby the current anomalous readings of a pair of sensors are also located within the corresponding area of the rotated ellipse, this may result in a false alarm.

While some works [16,21] have considered the collusive scenario, further development is needed for when an attacker employs stealthy attacks that construct coherent attack signals (mimicking genuine data distributions and time series patterns). Therefore, in this paper, we propose a generalized detection framework that can be used to detect FDIAs in large-scale WSNs, including stealthy FDIAs. The approach we propose in this paper to meet these requirements, together with the previously mentioned works, is summarized in Table 1.

## 3. Preliminaries

We extract information from the sensor data itself to detect FDIAs. In this section, we discuss the definition of sensor data and the correlation between sensor data.

### 3.1. Sensor Data

Consider a set of sensors $V=\{v_{1},\cdots ,v_{N}\}$ distributed over a geographic area, where each sensor $v_{i}\in V$ collects one type of environmental data in synchronization with the other sensors. Let $r_{i}\left(t\right)$ denote the sensor measurement reported by $v_{i}$ at time *t*, as follows:

(1) $$r_{i}\left(t\right)=\tilde{r_{i}}\left(t\right)+\epsilon \left(t\right),$$

where $\tilde{r_{i}}\left(t\right)$ is the true value and ϵ(t) is an error at time *t*. The error ϵ(t) can be caused by either a random error or a systematic error. A random error is an uncertainty in the measurement result caused by various random factors (e.g., noise), and a systematic error is an uncertainty in the measurement result due to inherent defects or biases (e.g., faults, FDIAs). Since our work focuses on detecting FDIAs on sensors, we only consider systematic errors caused by FDIAs. The collection of $r_{i}\left(t\right)$ from $v_{i}$ over a period of time is a time-series signal [25]. A time-series signal consisting of *t* successive sensor measurements $r_{i}\left(1\right),r_{i}\left(2\right),\cdots ,r_{i}\left(t\right)$ can be expressed as follows:

(2) $$R_{i}\left(t\right)=\left\{r_{i}\left(1\right),r_{i}\left(2\right),\cdots ,r_{i}\left(t\right)\right\}.$$

### 3.2. Spatiotemporal Correlation between Sensor Data

Spatiotemporal correlation is a combination of spatial and temporal correlation, referring to the simultaneous existence of correlations in space and time. The correlation of sensor data exists because sensors are distributed in space and measure time-dependent physical phenomena. The anomalous data generated when an FDIA occurs can go so far as to cause this correlation to be disrupted, so we can identify false data injection attacks by analyzing the correlation of sensor data [26].

#### 3.2.1. Spatial Correlation

Spatial correlation between sensor data over a fixed time interval reveals the degree of association between events or phenomena at adjacent or discrete locations in space. For example, in a smart grid, neighboring industrial facilities may belong to similar industries and, thus, have a similar electricity demand, resulting in a strong spatial correlation between meter data in industrial areas, but there may be a weak spatial correlation between meter data in industrial areas and meter data in residential areas.

#### 3.2.2. Temporal Correlation

The temporal correlation of sensor data reveals the degree of association between events or phenomena over time. For example, in a smart grid, due to differences between day and night, seasonal factors, etc., by observing hourly, daily, weekly, or seasonal data from meters, it is possible to find repeating patterns or regularities in the use of electrical energy on different time scales.

## 4. FDIA Detection Framework

In this section, this paper proposes a framework for FDIA detection. This framework consists of three phases: correlation grouping, correlation prediction, and correlation testing, stated as follows:

- *Phase I: Correlation grouping*. The purpose of this phase is to group *V* in a large-scale WSN based on historical sensor data so that sensors in the same group are highly correlated with other sensors.
- *Phase II: Correlation prediction*. The purpose of this phase is to predict the normal temporal correlation of the cross-correlation between pairwise sensor measurements in the same group over a short period of time in the future.
- *Phase III: Correlation testing*. The purpose of this phase is to test the actual sensor data based on the predicted normal temporal and spatial correlations.

**Figure 1:**
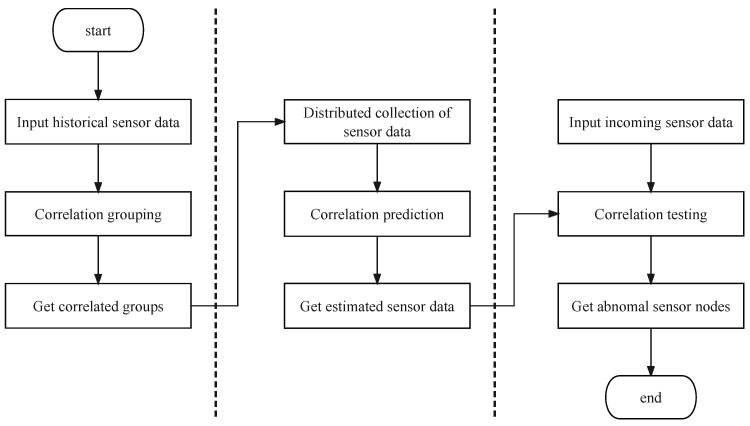
The flow diagram for FDIA detection in large-scale WSNs.

The flow diagram for FDIA detection in large-scale WSNs is shown in Figure 1. Next, let us discuss the three phases in detail.

### 4.1. Correlation Grouping

Collect sensor data, ensuring that the data are collected at the same or similar frequencies, and pre-process the data if necessary, including de-noise, filling in missing values, interpolating, and other operations to facilitate analysis. Standardize the sensor data (e.g., min-max normalization, z-score normalization) to ensure that the measurements from different sensors are similarly scaled so that the magnitude of the change in one sensor does not affect the cross-correlation results.

Let $R_{i}\left(T\right)=\left\{r_{i}\left(1\right),r_{i}\left(2\right),\cdots ,r_{i}\left(T\right)\right\}$ denote the Historical Time-series Signal (HTS) of $v_{i}$ obtained after data processing, where *T* denotes the length of the HTS. The cross-correlation of any two full HTSs is usually calculated to determine the spatial correlation between $R_{i}\left(T\right)$ and $R_{j}\left(T\right)$, expressed as follows:

(3) $$C_{ij}=\frac{cov(R_{i}\left(T\right),R_{j}\left(T\right))}{\delta \left(R_{i}\left(T\right)\right)\delta \left(R_{j}\left(T\right)\right)},$$

where

(4) $$cov\left(R_{i}\left(T\right),R_{j}\left(T\right)\right)=\frac{1}{T}\sum_{t=1}^{T}\left(r_{i}\left(t\right)-\bar{r_{i}}\right)\left(r_{j}\left(t+\tau \right)-\bar{r_{j}}\right)$$

denotes the covariance between $R_{i}\left(T\right)$ and $R_{j}\left(T\right)$, $\delta \left(R_{i}\left(T\right)\right)=\sqrt{\frac{1}{T}\sum_{t=1}^{T}{\left(r_{i}\left(t\right)-\bar{r_{i}}\right)}^{2}}$, and $\delta \left(R_{j}\left(T\right)\right)=\sqrt{\frac{1}{T}\sum_{t=1}^{T}{\left(r_{j}\left(t+\tau \right)-\bar{r_{j}}\right)}^{2}}$ denote the standard deviations of $R_{i}\left(T\right)$ and $R_{j}\left(T\right)$, respectively. $C_{ij}$ denotes the correlation coefficients of $v_{i}$ and $v_{j}$ at lag τ; $\bar{r_{i}}$ and $\bar{r_{j}}$ represent the average values of two full HTSs from $v_{i}$ and $v_{j}$. The lag τ represents the delay of one HTS with respect to the other, and by analyzing the peak of cross-correlation, it is determined at which lag value the correlation between the two HTSs is greatest. $C_{ij}$ has a value between 1 and −1, where 1 means perfect positive correlation, −1 means perfect negative correlation, and 0 means the signals are uncorrelated [27].

However, this paper’s goal is to extract the temporal correlation of the cross-correlation between any two HTSs, so the sliding window cross-correlations need to be computed.

First, let the size of the sliding window be *k*. The *w*th sub-signal, consisting of *k* successive sensor measurements $r_{i}\left(w\right),\cdots ,r_{i}\left(w+k-1\right)$ within [1,t], can be defined as

(5) $$R_{i}\left(t,w\right)=\left\{r_{i}\left(w\right),\cdots ,r_{i}\left(w+k-1\right)\right\}.$$

Therefore, the HTS of $v_{i}$ is segmented into multiple *historical sub-signals*, denoted as $R_{i}=\left\{R_{i}\left(T,1\right),R_{i}\left(T,2\right),\cdots ,R_{i}\left(T,W\right)\right\}$, where *W* denotes the number of historical sub-signals.

Second, for $R_{i}$ and $R_{j}$, the cross-correlation is computed within each sliding window, denoted as

(6) $$C_{ij}\left(w\right)=\frac{cov(R_{i}\left(T,w\right),R_{j}\left(T,w\right))}{\delta \left(R_{i}\left(T,w\right)\right)\delta \left(R_{j}\left(T,w\right)\right)},\;w=1,2,\cdots ,W.$$

Here, $cov(R_{i}\left(T,w\right),R_{j}\left(T,w\right))$ represents the covariance between $R_{i}\left(T,w\right)$ and $R_{j}\left(T,w\right)$; $\delta (R_{i}\left(T,w\right))$ and $\delta (R_{j}\left(T,w\right))$ represent the standard deviations of $R_{i}\left(T,w\right)$ and $R_{j}\left(T,w\right)$, respectively. Then, the time series of the cross-correlation of $v_{i}$ and $v_{j}$ can be represented by

(7) $$C_{ij}=\left\{C_{ij}\left(1\right),C_{ij}\left(2\right),\cdots ,C_{ij}\left(W\right)\right\},\;w=1,2,\cdots ,W.$$

Finally, we pick $C_{ij}$ with a positive correlation for *K*-means clustering, which is one of the most widely used parameter selection methods. After *K*-means clustering, the sensors can be categorized into multiple correlation groups.

For a dataset with *M* time series of cross-correlation, we represent $C_{ij}$ with a positive correlation as a feature vector. We extract relevant features that capture the characteristics of the time series; commonly used features include mean, standard deviation, slope, etc. Each $C_{ij}$ with a positive correlation is represented as a feature vector $v_{p}=\left[f_{1},f_{2},\cdots ,f_{k}\right]$ (p=1,⋯,M), where *k* is the number of features and $v_{p}$ includes all necessary extracted features (mean, standard deviation, slope, etc.). The random cluster centers $u_{1},u_{2},\cdots ,u_{K}$ are first selected, and then the *K*-means objection function is defined as follows:

(8) $$J=\sum_{p=1}^{M}\sum_{q=1}^{K}x_{pq}\cdot {∥v_{p}-u_{q}∥}^{2},$$

where $x_{pq}$ is an indicator function indicating if the time series *p* belongs to cluster *q* (q=1,2,⋯,K), and ${∥\cdot ∥}^{2}$ denotes the squared Euclidean distance.

We update the centroids of the clusters by calculating the mean feature vector for each cluster:

(9) $$u_{q}=\frac{1}{\left|C_{q}\right|}\sum_{p\in C_{q}}v_{p},$$

where $\left|C_{q}\right|$ is the number of time series in cluster *q*. We repeat the centroid’s update and minimization of *J* until convergence. Then,

**Definition** **1.** *Let $V_{i}^{q}=\left\{v_{j}|C_{ij}\in clustr\;q,j\neq i\right\}$ be the set of sensors consistent with sensor $v_{i}$ in cluster q obtained according to HTSs, and let $V^{q}=\left\{v_{i}|\frac{|V_{i}^{q}|}{N-1}>50\%\right\}$ be the set of sensors that are grouped in q according to HTSs.*

**Figure 2:**
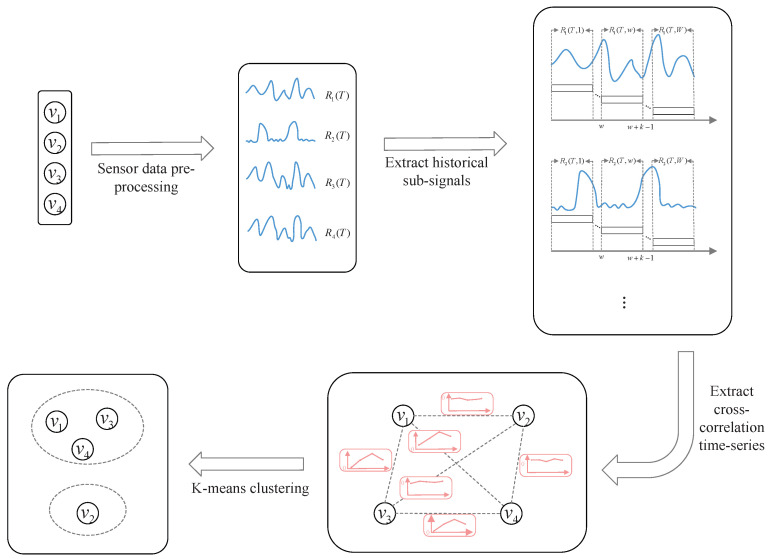
The correlation grouping of four sensor nodes.

**Remark** **1.** *Figure 2 illustrates the correlation grouping of four sensor nodes. After correlation grouping, each group’s sensor data can be sent to a separate edge device for distributed processing to reduce network pressure and improve processing efficiency [28]. The following stages are performed within each group: correlation prediction and correlation testing.*

### 4.2. Correlation Prediction

Next, we predict the normal temporal correlation of cross-correlation between pairwise sensor measurements in each group over a short period of time in the future.

Consider pairwise sensors $v_{i}$ and $v_{j}$ in a group. As we discussed in the previous subsection, the measurements of $v_{i}$ and $v_{j}$ should be temporally correlated with their previous measurements. Therefore, this subsection uses the Autoregressive Integrated Moving Average (ARIMA) model to predict the future time-series signal of each sensor based on the HTS, which is referred to as the Estimated Time-series Signal (ETS). ARIMA is used as a time series predictive analysis method, which requires only historical data to make predictions and has the ability to be widely applied to a wide range of time series data.

ARIMA combines the concepts of autoregression (AR), moving average (MA), and the operation of differencing the time series signals. Specifically, the autoregressive part represents the relationship between the current value of a variable and its value at $p^{\prime }$ previous moments, where $p^{\prime }$ denotes the autoregressive order. The moving average part represents the relationship between the current value and the error (white noise) at $q^{\prime }$ previous moments, where $q^{\prime }$ denotes the moving average order. The *d*-order differencing operation is performed to remove trends and seasonality from HTSs. Therefore, an ARIMA model is used to fit the trend and periodicity of the HTS by choosing appropriate parameters $\left(p^{\prime },d,q^{\prime }\right)$ to make forecasts of the ETS [29].

First, a suitable *d* is chosen using the following difference method:

(10) $$\Delta^{d}r_{i}\left(t\right)=\Delta \left(\Delta^{d-1}r_{i}\left(t\right)\right),$$

where $\Delta r_{i}\left(t\right)=r_{i}\left(t\right)-r_{i}\left(t-1\right)$ denotes the first-order difference at time point *t*. The suitable value is *d* when the sequence after *d*-order differencing of the HTS passes the Augmented Dickey–Fuller (ADF) test [30].

Second, for all possible combinations of $p^{\prime }$ and $q^{\prime }$, an ARIMA model is fitted using the information criterion (AIC) to select the best combination of $p^{\prime }$ and $q^{\prime }$ as the one with the smallest AIC value. The formula for calculating AIC is as follows:

(11) AIC=−2ln (L)+2l,

where *L* is the maximum likelihood estimate of the model, and *l* is the number of parameters of the model.

Third, the HTS is fitted using an ARIMA model with order $\left(p^{\prime },d,q^{\prime }\right)$, which is formulated as follows:

(12) $$\Delta^{d}r_{i}\left(t\right)=\mu +\sum_{l=1}^{p^{\prime }}\phi_{l}\Delta^{d}r_{i}\left(t-l\right)+\sum_{l=1}^{q^{\prime }}\psi_{l}\epsilon \left(t-l\right)+\epsilon \left(t\right),$$

where μ, $\phi_{l}$, and $\psi_{l}$ are model parameters, and ϵ(t) stands for the value of the independent error at time *t*, which follows a Gaussian distribution with a zero mean. The fitted model is tested to see if it matches the characteristics of the data, including the autocorrelation and partial autocorrelation of the residuals and normality of the residuals.

Finally, assuming that the fitted model is used to predict future data, the ETSs can be made by the difference restoration of predicted data. An estimated time-series signal consisting of *t* successive sensor measurements $r_{i}^{\prime }\left(1\right),r_{i}^{\prime }\left(2\right),\cdots ,r_{i}^{\prime }\left(t\right)$ can be expressed as follows:

(13) $$R_{i}^{\prime }\left(t\right)=\left\{r_{i}^{\prime }\left(1\right),r_{i}^{\prime }\left(2\right),\cdots ,r_{i}^{\prime }\left(t\right)\right\}.$$

Then, let $R_{i}^{\prime }\left(S\right)=\left\{r_{i}^{\prime }\left(1\right),r_{i}^{\prime }\left(2\right),\cdots ,r_{i}^{\prime }\left(S\right)\right\}$ denote the ETS of $v_{i}$, where *S* denotes the length of the ETS.

The ETS and HTS are concatenated into a new time-series signal $X_{i}\left(t\right)$, which consists of *t* successive sensor measurements and can be expressed as follows:

(14) $$X_{i}\left(t\right)=\left\{\begin{array}{c} R_{i}\left(t\right),\;t\leq T,\\ \{R_{i}\left(T\right),R_{i}^{\prime }\left(t-T\right)\},\;t>T. \end{array}\right.$$

Similar to Equation (6), the *w*th *estimated sub-signal* consisting of *k* successive sensor measurements within [1,t] can be defined as $X_{i}\left(t,w\right)$. $C_{ij}^{\prime }=\left\{C_{ij}\left(1\right),C_{ij}\left(2\right)\cdots ,C_{ij}\left(S\right)\right\}$ is calculated in the same manner as in Equation (7) based on $X_{i}\left(t,w\right)$ and $X_{j}\left(t,w\right)$, where w=1,2,⋯,S.

**Figure 3:**
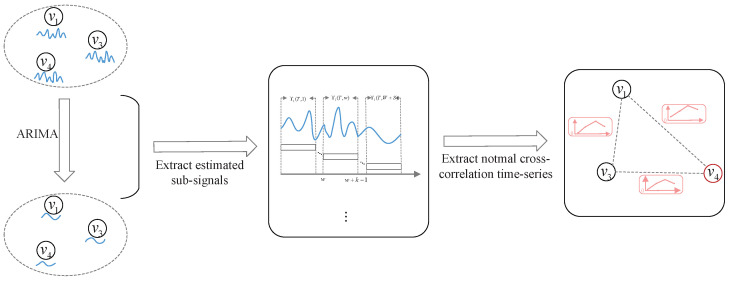
The diagram of correlation prediction within a group.

Therefore, $C_{ij}^{\prime }$ represents the normal temporal correlation of cross-correlation between pairwise sensor measurements in a group. The diagram of correlation prediction within a group is shown in Figure 3.

### 4.3. Correlation Testing

After correlation prediction, we compare $C_{ij}^{\prime }$ with the actual ones to detect FDIAs in this subsection.

An actual time-series signal consisting of *t* successive sensor measurements $r_{i}^{*}\left(1\right),r_{i}^{*}\left(2\right),$$\cdots ,r_{i}^{*}\left(t\right)$ can be expressed as follows:

(15) $$R_{i}^{*}\left(t\right)=\left\{r_{i}^{*}\left(1\right),r_{i}^{*}\left(2\right),\cdots ,r_{i}^{*}\left(t\right)\right\}.$$

Then, let $R_{i}^{*}\left(S\right)=\left\{r_{i}^{*}\left(1\right),r_{i}^{*}\left(2\right),\cdots ,r_{i}^{*}\left(S\right)\right\}$ denote the Actual Time-series Signal (ATS) of $v_{i}$, where *S* denotes the length of the ATS.

The ATS and HTS are concatenated into a new time-series signal $Y_{i}\left(t\right)$, which consists of *t* successive sensor measurements and can be expressed as follows:

(16) $$Y_{i}\left(t\right)=\left\{\begin{array}{c} R_{i}\left(t\right),\;t\leq T,\\ \{R_{i}\left(T\right),R_{i}^{*}\left(t-T\right)\},\;t>T. \end{array}\right.$$

Similar to Equation (6), the *w*th *actual sub-signal*, which consists of *k* successive sensor measurements within [1,t], can be defined as $Y_{i}\left(t,w\right)$. Consider each pair of $v_{i}$ and $v_{j}$ in a group; $C_{ij}^{*}=\left\{C_{ij}\left(1\right),C_{ij}\left(2\right),\cdots ,C_{ij}\left(S\right)\right\}$ is calculated in the same manner as in Equation (7) based on $Y_{i}\left(t,w\right)$ and $Y_{j}\left(t,w\right)$, where w=1,2,⋯,S.

Therefore, $C_{ij}^{*}$ represents the actual temporal correlation of cross-correlation between pairwise sensor measurements in a group.

Consider each pair of $v_{i}$ and $v_{j}$ in the group q⊆V. Based on $C_{ij}^{\prime }$ and $C_{ij}^{*}$, we have

(17) $$\rho_{ij}=\frac{cov(C_{ij}^{\prime },C_{ij}^{*})}{\delta \left(C_{ij}^{\prime }\right)\delta \left(C_{ij}^{*}\right)},$$

where $cov(C_{ij}^{\prime },C_{ij}^{*})$ is the covariance of $C_{ij}^{\prime }$ and $C_{ij}^{*}$; $\delta (C_{ij}^{\prime })$ and $\delta (C_{ij}^{*})$ are the standard deviations of $C_{ij}^{\prime }$ and $C_{ij}^{*}$, respectively. Then, we have

(18) $$e_{ij}=\left\{\begin{array}{c} 1,\;if\rho_{ij}>\theta ,\\ 0,\;otherwise, \end{array}\right.$$

where $e_{ij}$ is for the *consistency criterion*, and $e_{ij}=1\left(resp.0\right)$ denotes that $v_{i}$ and $v_{j}$ are consistent (resp. inconsistent).

**Remark** **2.** *The choice of threshold θ depends on the experience of $C_{ij}$ in Phase I, and the performance of the model on the test set can be observed by trying different thresholds and selecting the one with the best performance.*

**Definition** **2.** *Let $N_{i}^{a}=\left\{v_{j}\right|e_{ij}=1$ or $e_{ij}=0,i\neq j\}$ be the set of all neighbors of $v_{i}$, and $N_{i}^{c}=\left\{v_{j}|e_{ij}=1,i\neq j\right\}$ be the set of consistent neighbors of $v_{i}$ obtained according to the comparison of ETSs and ATSs.*

**Definition** **3.** *Let $N^{t}=\left\{v_{i}\in q|\frac{|N_{i}^{c}|}{|N_{i}^{a}|}\geq 50\%\right\}$ be the set of all trusted neighbors.*

**Figure 4:**
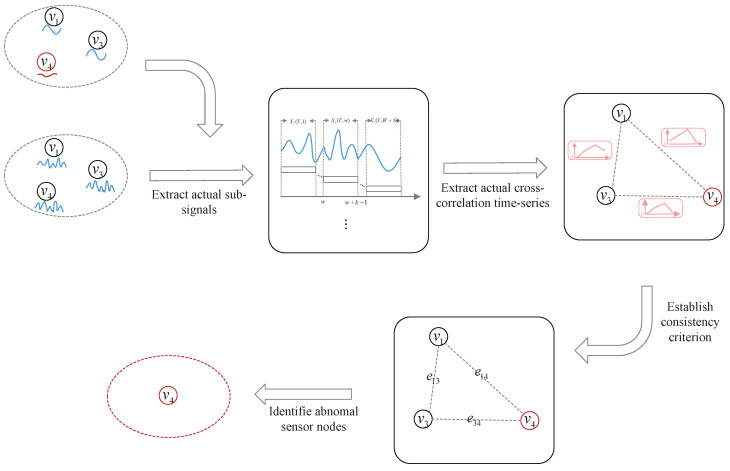
The diagram of correlation testing within a group.

So, let $N^{F}=\left\{v_{i}\in q\right|\frac{|N^{t}⋂N_{i}^{a}|}{|N_{i}^{a}|}\geq 50\%$ and $\frac{|N^{t}⋂N_{i}^{a}⋂N_{i}^{c}|}{|N^{t}⋂N_{i}^{a}|}<50\%\}$ be the set of abnormal nodes in group *q*. The diagram of correlation testing within a group is shown in Figure 4.

## 5. Effectiveness of the Proposed Framework

This section is devoted to investigating the effectiveness of the framework through simulation experiments.

### 5.1. Experiment Preparation

We applied the detection framework to an hourly electricity demand dataset by subregion, which was based on the 2020 US Energy Information Administration State Electricity Profiles (available at http://www.eia.gov/, accessed on 2 June 2023). This dataset was chosen because it was derived from widely distributed smart meters to evaluate the effectiveness of our proposed framework in detecting FDIAs.

By visualizing the dataset, we found that the time series data show a pronounced periodicity with a period length of 24. Therefore, the model parameters used for this dataset were obtained through observation and manual grid search, as shown in Table 2.

### 5.2. Experiments and Analysis of Experimental Results

**Figure 5:**
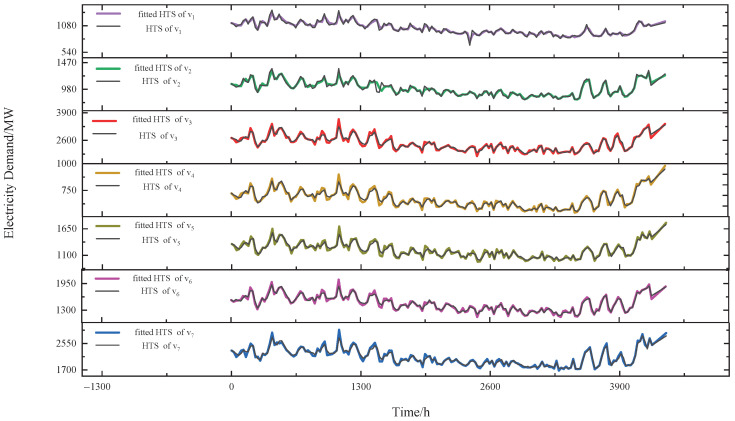
The results for one of the groups after correlation grouping and data fitting for HTSs.

Figure 5 illustrates the results for one of the groups, consisting of a set of sensors $\left\{v_{1},v_{2},\cdots ,v_{7}\right\}$, after correlation grouping and data fitting for HTSs. In Figure 5, we visualize only the data points with a step size of 24 to display the fitting results clearly. As can be seen from the figure, there is a strong correlation between the HTSs within a group, and our approach effectively fits the HTSs.

**Figure 6:**
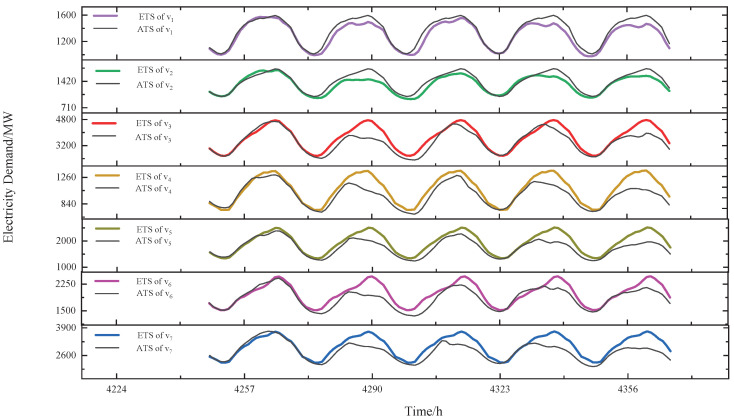
The comparison results of ETSs and ATSs for the group after correlation prediction.

Figure 6 illustrates the comparison results of ETSs and ATSs for the group after correlation prediction. It is seen that our approach can effectively predict future data.

To further validate the effectiveness of our framework for detecting FDIAs, we performed correlation testing for various FDIA strategies on target signals. Moreover, we compared our approach with the SCCR solution given in previous work [18], where the SCCR is a consistent ellipse area formed by spatiotemporal correlations. In our experiments, the confidence degree of the consistency ellipse was set to 95%.

In addition, we used three different metrics: successful detection rate, false-negative detection rate, and false-positive detection rate. The successful detection rate is the proportion of actual abnormal nodes that are correctly identified; the false-negative detection rate is the proportion of actual abnormal nodes that are incorrectly identified as normal; and the false-positive detection rate is the proportion of actual normal nodes that are incorrectly identified as abnormal nodes.

#### 5.2.1. The Simple FDIA

**Figure 7:**
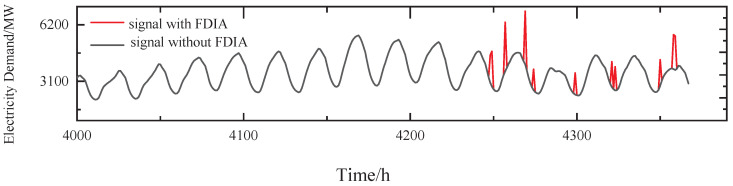
The simple FDIA on $v_{3}$.

A simple FDIA means randomly generating an attack signal. Assuming that $r_{3}\left(t\right)$ is chosen as the target of the attack of the group, the power demand of $v_{3}$ is randomly increased by 50%, as shown in Figure 7.

**Figure 8:**
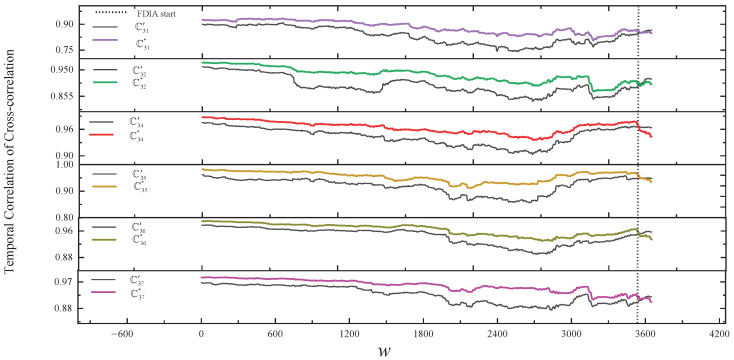
The results of comparing the normal temporal correlation of cross-correlation with the actual temporal correlation of cross-correlation of $v_{3}$ within the group in a simple FDIA.

In our solution, Figure 8 and the second line of Table 3 show the results of comparing the normal temporal correlation of cross-correlation with the actual temporal correlation of cross-correlation of $v_{3}$ within the group in a simple FDIA. As shown in Figure 8, from the start of the FDIA, the change in the trend of $C_{ij}^{*}$ relative to the trend of $C_{ij}^{\prime }$ is clearly inconsistent. In the SCCR solution, we can also observe the inconsistency of $v_{3}$ with other nodes. The proposed framework and SCCR solution are able to accurately detect the simple FDIA on $v_{3}$.

**Figure 9:**
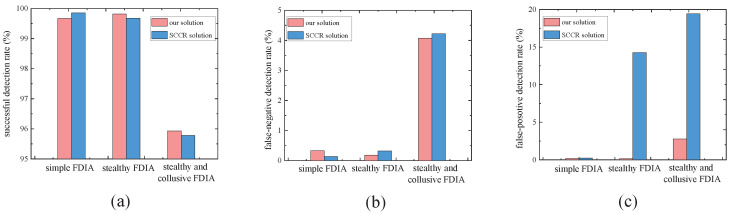
The comparison results of three metrics: (**a**) successful detection rate; (**b**) false-negative detection rate; (**c**) false-positive detection rate.

We conducted a total of 100 similar experiments in all groups, in which the framework proposed in this paper and the SCCR solution were able to detect at least 99% of FDIAs (Figure 9a), and the false-negative detection rate (Figure 9b) and false-positive detection rate (Figure 9c) were almost zero. Therefore, we conclude that, in general, the framework proposed in this paper performs well in detecting simple FDIAs.

#### 5.2.2. The Stealthy FDIA

**Figure 10:**
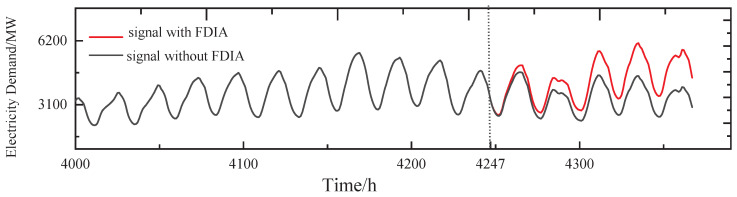
The stealth FDIA on $v_{3}$.

The stealthy FDIA means the attacker injects in a well-designed way that is generally not easily observable. Assuming that $r_{3}\left(t\right)$ is chosen as the target of the attack and that the attacker is able to learn the time series of $v_{3}$ of the group, in this case, the power demand of $v_{3}$ slowly increases within the detected threshold (boiling frog attack [31]) and also exhibits periodicity from t=4247 h, as shown in Figure 10.

**Figure 11:**
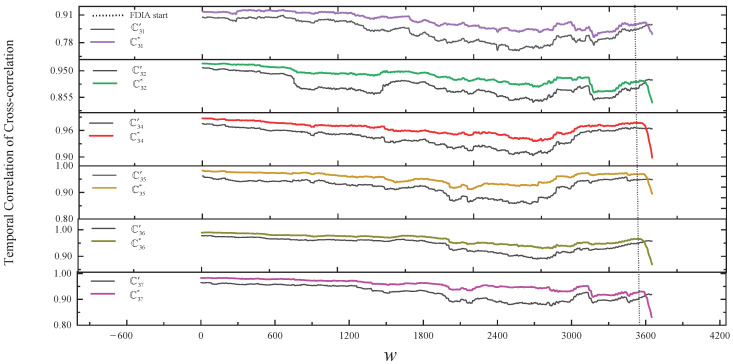
The results of comparing the normal temporal correlation of cross-correlation with the actual temporal correlation of cross-correlation of $v_{3}$ within the group in a stealthy FDIA.

In our solution, Figure 11 and the third line of Table 3 show the results of comparing the normal temporal correlation of cross-correlation with the actual temporal correlation of cross-correlation of $v_{3}$ within the group in stealthy FDIA. As shown in Figure 11, from the start of the FDIA, the change in the trend of $C_{ij}^{*}$ relative to the trend of $C_{ij}^{\prime }$ is gradually inconsistent. However, in the SCCR solution, we do not identify any outliers during the first 66 h of the FDIA, after which the abnomal nodes $v_{3}$, $v_{6}$, and $v_{7}$ are identified. This result is caused by the fact that at the beginning of the FDIA, the outliers are within the detection threshold of the SCCR solution, leading to unrecognized anomalies, which are then considered normal to build the consistency ellipse, resulting in a high rate of false positives.

We conducted a total of 100 similar experiments in all groups, in which both the framework proposed in this paper and the SCCR solution were able to detect at least 99% of FDIAs (Figure 9a), and the false-negative detection rate was almost zero (Figure 9b); the false-positive detection rate for the framework proposed in this paper was almost zero, while the false-positive detection rate for the SCCR solution was up to 14% (Figure 9c). In addition, we observed that long-term attack signals resulted in stronger inconsistencies than short-term attack signals. Therefore, we conclude that, in general, the framework proposed in this paper performs well for detecting stealthy FDIAs on a single sensor, and our approach is superior compared to the SCCR solution in detecting long-term and stealthy attack signals. However, the inconsistency was not obvious from the beginning of the FDIA. Therefore, it is necessary to choose a suitable ETS size or sliding window size when detecting stealthy FDIAs.

**Figure 12:**
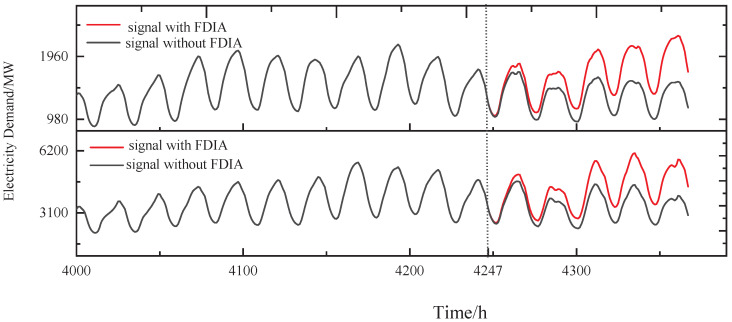
The stealthy and collusive FDIA on $v_{2}$ and $v_{3}$.

In addition, assuming there is a collision, the attacker chooses the next node whose data are consistent with node $v_{3}$ as the next attack target to work in concert. With $v_{2}$ chosen as the next attack target, the same FDIA strategy is used to construct an attack signal for $v_{2}$ after the attacker learns the cross-correlation between $v_{2}$ and $v_{3}$. Figure 12 shows the signals of $v_{2}$ and $v_{3}$ with FDIA and without FDIA, where the FDIA starts at t=4247 h.

**Figure 13:**
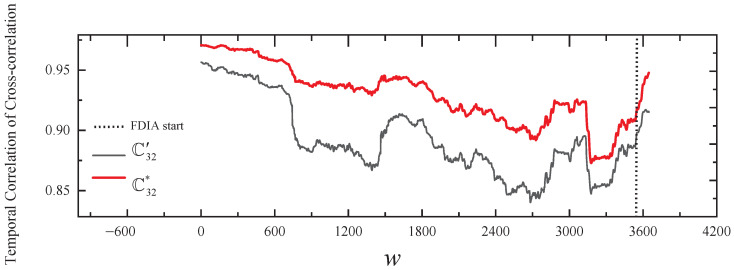
The results of comparing the normal temporal correlation of cross-correlation with the actual temporal correlation of cross-correlation of $v_{2}$ and $v_{3}$ in stealthy and collusive FDIAs.

In our solution, Figure 13 and the fourth line of Table 3 show the results of comparing the normal temporal correlation of cross-correlation with the actual temporal correlation of cross-correlation of $v_{2}$ and $v_{3}$ within the group in stealthy and collusive FDIAs. As shown in Figure 13, from the FDIA’s start, the change in the trend of $C_{32}^{*}$ relative to the trend of $C_{32}^{\prime }$ is relatively consistent, and $\rho_{32}=0.98$ also indicates that the readings of the collusive nodes are consistent. Due to the proposed voting algorithm, the framework can still detect the stealthy and collusive FDIAs on $v_{2}$ and $v_{3}$. However, in the SCCR solution, we similarly do not identify any outliers during the first 66 h of the FDIA, after which the abnormal nodes $v_{2}$, $v_{3}$, $v_{6}$, and $v_{7}$ are identified.

We conducted a total of 100 similar experiments in all groups, in which both the framework proposed in this paper and the SCCR solution were able to detect at least 95% of FDIAs (Figure 9a) with no more than a 5% false-negative detection rate (Figure 9b); the false-positive detection rate for the framework proposed in this paper was, again, no more than 3%, while the false-positive detection rate for the SCCR solution was as high as 19% (Figure 9c). Furthermore, we observed that long-term attack signals result in stronger inconsistencies than short-term attack signals. Therefore, we conclude that, in general, the framework proposed in this paper performs well for detecting stealthy FDIAs in two collusive sensors, and our approach is superior compared to the SCCR solution in detecting long-term, stealthy, and collusive attack signals. However, as the number of collusive sensors increased, we observed a performance degradation. The proposed detection algorithm fails when the number of collusive sensors exceeds 50%. This is due to the fact that the detection algorithm uses majority voting, and more than 50% of the sensors must be normal to ensure the performance of the detection.

Overall, the framework proposed in this paper performs best in detecting simple and stealthy FDIAs in single-sensor scenarios and is relatively effective in detecting stealthy FDIAs in multi-sensor scenarios.

## 6. Conclusions and Future Works

This paper presents a novel detection framework for FDIAs on large-scale WSNs. The framework consists of three phases. The first stage groups the sensors, which is based on the temporal correlation of the cross-correlation between the pairwise sensors. The second phase proposes a model for learning the temporal correlation of the cross-correlation. The third stage establishes consistency criteria within each group and votes out the abnormal nodes. We validated the performance of the framework by simulating simple FDIAs and stealthy FDIAs on a real dataset.

However, the detection framework also has some limitations. First, this paper only considers the scenario where FDIAs exist, and the framework is not designed to distinguish between FDIAs and natural anomalies, disruptive events, etc. Second, ARIMA is usually more suitable for forecasting problems with one-dimensional time series data, while for more complex problems, especially when multidimensional data are involved, the method needs to be further optimized. In addition, the voting algorithm fails to detect FDIAs on more than 50% of the sensors, and there is merit in exploring detection methods in the collusion-tolerant anomaly. Thus, there is value in further research on an anomaly score aggregation that tolerates collusion, and future work on the detection framework can be optimized by exploring other techniques to distinguish between FDIAs and natural anomalies. In addition, using a distributed detection framework that takes into account the trade-off between cost and criticality, the work can be conducted in the context of an optimization problem, such as the allocation of defense resources [32,33]. Finally, the framework proposed in this paper can be generalized to other correlation-based problems, such as advanced persistent threat detection [34,35], DDoS detection [36,37], and event-triggered state estimation [38,39].

## Figures and Tables

**Table 1:** Comparison between approaches.

Research Works	FDIA Detection Methods		FDIA Types		
	General Detection	Distributed Detection	Simple FDIAs	Collusive FDIAs	Stealthy FDIAs
Illiano et al. [16]	yes	no	yes	yes	no
Aboelwafa et al. [17]	yes	no	yes	no	no
Martovytskyi et al. [18]	yes	no	yes	no	no
Berjab et al. [19]	yes	no	yes	no	no
Huang et al. [20]	yes	no	yes	no	no
Hu et al. [21]	yes	no	yes	yes	no
Chen et al. [22]	yes	yes	yes	no	no
Islam et al. [23]	yes	yes	yes	no	no
Lai et al. [24]	yes	yes	yes	no	no
Our approach	yes	yes	yes	yes	yes

**Table 2:** Model parameters used on the dataset.

Parameters	Value
The HTSs’ size *T*	4246 h
The ETSs’ size *S*	120 h
Sliding window size *k*	720 h
Threshold θ	0

**Table 3:** The comparison results of the temporal correlation of cross-correlation.

Fdia Type	$\rho_{31}$	$\rho_{32}$	$\rho_{34}$	$\rho_{35}$	$\rho_{36}$	$\rho_{37}$
Simple FDIA	−0.18	0.15	0.10	−0.48	−0.66	−0.44
Stealthy FDIA	−0.60	−0.67	0.03	−0.21	−0.79	−0.71
Stealthy and collusive FDIA	−0.60	0.98	0.03	−0.21	−0.79	−0.71

## Data Availability

Data are available upon request.
